# Impact of the COVID-19 pandemic on diagnosis, treatment, and prognosis of cervical, endometrial, and ovarian cancers: A retrospective cohort study based on the SEER database

**DOI:** 10.1097/MD.0000000000051038

**Published:** 2026-10-16

**Authors:** Chunyan Tan, Huibo Dai, Xingya Liu, Ning Xu, Xinli Zhong, Yanqiu Liu

**Affiliations:** a The First People’s Hospital of Shuangliu District, Chengdu City/West China Airport Hospital of Sichuan University, Chengdu, Sichuan, China.

**Keywords:** cancer screening disruption, COVID-19 pandemic, gynecologic cancers, socioeconomic disparities, stage migration

## Abstract

The coronavirus disease 2019 pandemic disrupted healthcare systems worldwide, but population-based evidence on its effects on gynecologic cancer care remains limited. We evaluated changes in diagnosis, disease presentation, treatment timeliness, and cancer-specific survival (CSS) for cervical, endometrial, and ovarian cancers during the pandemic. In this retrospective cohort study, Surveillance, Epidemiology, and End Results (SEER)-22 data were used to evaluate trends in newly diagnosed cases from 2017 to 2021, and SEER-17 data to compare patients diagnosed in 2020 (pandemic cohort) with those diagnosed in 2018 to 2019 (pre-pandemic cohort). Eligible patients were aged 20 to 85 years with primary cervical, endometrial, or ovarian cancer. Demographic characteristics, disease presentation, time from diagnosis to treatment initiation, and CSS were compared using ordinal logistic regression, logistic regression, and Kaplan–Meier/log-rank analyses. The cohorts comprised 7997 patients with cervical, 36,151 with endometrial, and 11,508 with ovarian cancer; median follow-up in the pandemic cohort was 27 to 28 months. In SEER-22, new diagnoses in 2020 decreased by 8.7%, 5.5%, and 6.5% for cervical, endometrial, and ovarian cancers versus 2019. Diagnosis in 2020 was associated with more advanced-stage endometrial cancer (odds ratio, 1.06; 95% confidence interval [CI], 1.01–1.12; P = .027) but not cervical or ovarian cancer. Median time from diagnosis to treatment was shorter in the pandemic cohort for cervical (28 vs 30 days; *P* = .019) and endometrial cancer (28 vs 31 days; *P* < .001), but not for ovarian cancer (6 vs 4 days; *P* = .127). The pandemic cohort had borderline worse CSS for cervical cancer (hazard ratio [HR], 1.11; 95% CI, 1.00–1.24; *P *= .050) and worse CSS for endometrial cancer (HR, 1.10; 95% CI, 1.02–1.18; *P* = .009), but not ovarian cancer (HR, 1.05; 95% CI, 0.98–1.14; *P* = .185); 18-month CSS rates (pre-pandemic vs pandemic) were 86.6% vs 84.5%, 93.1% vs 92.6%, and 82.8% vs 83.0%. The pandemic period was associated with fewer recorded diagnoses of the 3 major gynecologic cancers, a modest shift toward more advanced-stage endometrial cancer, and worse short-term CSS for cervical and endometrial cancers. The reduction in recorded diagnoses may reflect disrupted screening and delayed presentation rather than a true decline in cancer occurrence.

## 1. Introduction

The coronavirus disease 2019 (COVID-19) pandemic in 2020 placed unprecedented strain on healthcare systems worldwide.^[1–3]^ Although lockdown measures implemented by governments globally achieved some success in controlling viral transmission, they also exerted substantial negative effects on the management of other diseases. For instance, these measures resulted in disruptions or delays in cancer screening services during the pandemic,^[4–6]^ restructuring of diagnostic and treatment workflows,^[7–9]^ and potential risks to patient survival outcomes.^[10–12]^ Despite these findings, most existing studies have focused on single cancer types or specific aspects of impact, lacking comprehensive assessments across multiple dimensions. Relatively short study periods have also limited the ability to capture potential long-term effects on prognosis. Furthermore, many investigations have relied on data from single clinical centers, thereby restricting the generalizability of findings to broader populations.

The Surveillance, Epidemiology, and End Results (SEER) program, supported by the National Cancer Institute, is a population-based cancer surveillance system that collects information on cancer incidence, patient demographics, tumor characteristics, first-course treatment, and survival from geographically diverse cancer registries in the United States.^[13]^ Its standardized data collection and broad population coverage make it a valuable resource for evaluating national patterns of cancer care. In the present study, two complementary SEER datasets were used. The SEER-22 dataset includes 22 registries and covers approximately 47.9% of the US population, providing the broadest available coverage for evaluating changes in the number of newly diagnosed cases. The SEER-17 dataset includes 17 registries and covers approximately 26.5% of the US population; it contains the detailed demographic, tumor, treatment, and survival variables required for adjusted analyses of stage at diagnosis, time to treatment initiation, and cancer-specific survival (CSS).^[13]^

Our objective was to comprehensively investigate the multidimensional impact of the COVID-19 pandemic in 2020 on 3 major gynecologic cancers, including cervical, endometrial, and ovarian cancers. Specifically, we analyzed the pandemic’s effects on the number of diagnosed cases, cancer stage at diagnosis, patient demographic characteristics, time interval from diagnosis to treatment initiation, and prognosis, aiming to derive insights from population-based data analysis.

## 2. Patients and methods

### 2.1. Data source and patient selection

This retrospective cohort study used data from the SEER program of the National Cancer Institute. Data were extracted from the SEER program using SEER*Stat software (National Cancer Institute). Trends in newly diagnosed cases from 2017 to 2021 were evaluated using the Incidence SEER Research Limited-Field Data, 22 Registries (excluding Illinois and Massachusetts), Nov 2023 Sub (2000–2021), released April 2024, extracted with SEER*Stat version 8.4.5; cohort analyses were conducted using the Incidence SEER Research Data, 17 Registries, Nov 2024 Sub (2000–2022), released April 2025, with follow-up through December 2022, extracted with SEER*Stat version 9.0.42. The SEER-22 dataset was used to evaluate temporal changes in the number of newly diagnosed cervical, endometrial, and ovarian cancers from 2017 to 2021. The SEER-17 dataset was used for cohort analyses of demographic characteristics, disease presentation, time from diagnosis to treatment initiation, and CSS among patients diagnosed from 2018 to 2020.

For the SEER-17 cohort analyses, eligible patients were aged 20 to 85 years and had a primary diagnosis of cervical, endometrial, or ovarian cancer. Patients diagnosed in 2020 were defined as the pandemic cohort, and those diagnosed in 2018 to 2019 were defined as the pre-pandemic cohort. These labels denote the calendar period of cancer diagnosis rather than individual severe acute respiratory syndrome coronavirus 2 (SARS-CoV-2) infection status, which is not available in SEER. Patients were excluded if they had missing information on cancer stage, demographic variables of interest (race, marital status, median household income, or rural-urban continuum), time from diagnosis to treatment, or follow-up information (cause of death or follow-up duration). In addition, 9 patients (3 with cervical cancer and 6 with endometrial cancer) whose time from diagnosis to treatment was top-coded as ”731 + days” in SEER were excluded from the treatment timeliness analysis. Cancers of the cervix, endometrium, and ovary were identified using the International Classification of Diseases for Oncology, Third Edition. The patient-selection process is shown in Figure 1. This study adhered to the Declaration of Helsinki. The requirement for institutional review board approval and informed patient consent was waived because all patient information had been de-identified.

**Figure 1.**
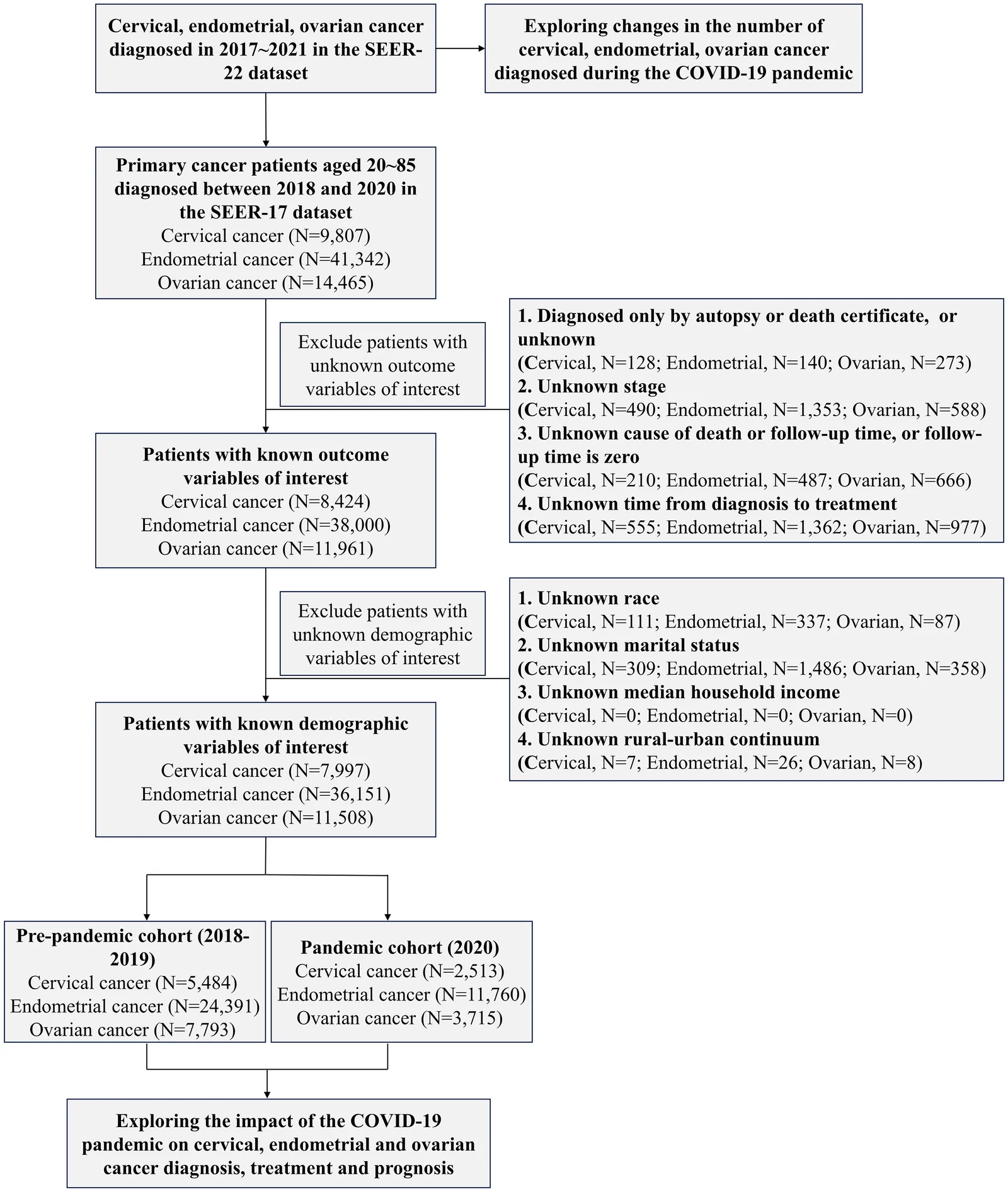
Flow diagram of patient selection. COVID-19 = coronavirus disease 2019.

### 2.2. Statistical analysis

Based on the SEER-22 dataset, we first described temporal trends in the number of newly diagnosed cases of the 3 major gynecologic cancers from 2017 to 2021. The Centers for Disease Control and Prevention announced the first case of severe acute respiratory syndrome coronavirus 2 (SARS-CoV-2) infection in the United States on January 20, 2020, marking the onset of the COVID-19 pandemic in the U.S.^[1–3]^ Therefore, we focused on changes in newly diagnosed cases in 2020 (the pandemic year) relative to 2019. Given that previous studies have demonstrated that the pandemic’s impact on cancer incidence varies by stage, we further analyzed changes in case numbers stratified by cancer stage.^[14]^

Using the SEER-17 dataset, we subsequently evaluated the impact of the COVID-19 pandemic in 2020 on the diagnosis, treatment, and prognosis of the 3 major gynecologic cancers. Patients were categorized into 2 groups based on year of diagnosis: those diagnosed in 2020 were assigned to the pandemic cohort, while those diagnosed in 2018 to 2019 served as the pre-pandemic cohort. Categorical variables were summarized as counts and percentages and compared using Pearson’s chi-square test or Fisher’s exact test, as appropriate. The distributions of continuous variables were assessed using the Anderson–Darling test and graphical methods, including histograms and quantile–quantile plots (Supplementary Figure S1, Supplemental Digital Content 1 and Supplementary Table S11, Supplemental Digital Content 2). Normally distributed continuous variables were summarized as means with standard deviations and compared using independent-samples t-tests; non-normally distributed continuous variables were summarized as medians with interquartile ranges and compared using Mann–Whitney *U* tests (equivalent to the Wilcoxon rank-sum test).

To assess the pandemic’s impact on cancer stage at diagnosis, cancer stage was analyzed as an ordinal outcome (Distant > Regional > Localized) using multivariable ordinal logistic regression, with year of diagnosis (2020 vs 2018–2019) as the primary explanatory variable. Cancer-specific models were fitted separately for the 3 cancers rather than combining them in a single model, given their distinct biological behavior, screening pathways, and stage distributions. Covariates were selected a priori according to clinical relevance, previous literature, and availability in SEER; the final models included age, race, marital status, median household income, rural-urban continuum, histological type, and tumor grade. Body mass index, comorbidities, performance status, and detailed referral information were unavailable in SEER and therefore could not be included. The proportional-odds assumption was assessed using the Brant test, and multinomial logistic regression was performed as a sensitivity analysis to verify the robustness of the ordinal models. Multicollinearity among covariates was assessed using generalized variance inflation factors. Unadjusted and adjusted odds ratios (OR), 95% confidence intervals (CI), and exact *P* values were reported, with the reference categories specified for all categorical variables.

Additionally, multivariable logistic regression was performed to explore predictors of diagnosis in 2020, thereby characterizing changes in the demographic profile of patients diagnosed during the pandemic. To evaluate the pandemic’s potential impact on treatment timeliness, time from diagnosis to treatment initiation was compared between the 2 cohorts using the Mann–Whitney *U* test; records with top-coded intervals (“731 + days”; n = 9) were excluded from this analysis. Finally, Kaplan–Meier survival curves and log-rank tests were used to assess the pandemic’s effect on CSS for the 3 cancers; hazard ratios (HR) were estimated using univariable Cox proportional hazards regression. All statistical analyses were conducted using R software (version 4.4.2). A two-sided *P* value < .05 was considered statistically significant.

## 3. Results

### 3.1. Decline in new cases during the COVID-19 pandemic

Figure 2 illustrates temporal trends in the number of newly diagnosed cases of the 3 major gynecologic cancers in the SEER-22 dataset from 2017 to 2021. From 2017 to 2019, the total number of new cervical and ovarian cancer cases remained relatively stable, whereas endometrial cancer cases demonstrated a year-overyear increase; however, all 3 cancers showed significant declines in new cases in 2020 compared with 2019 (Fig. 2A). Table 1 summarizes overall and stage-specific changes in 2020 relative to 2019 for these 3 cancers. Compared with 2019, total case numbers in 2020 decreased by 8.7% for cervical cancer, 5.5% for endometrial cancer, and 6.5% for ovarian cancer. Regarding stage-specific trends, new cases of localized and regional disease decreased in 2020 compared with 2019 across all 3 cancers (Figs. 2B–2C). For distant metastatic ovarian cancer, new cases also declined in 2020 (1.7%), but this decrease was markedly smaller than that observed for localized (12.6%) and regional (13.4%) disease (Fig. 2D). Conversely, new cases of distant metastatic cervical and endometrial cancers increased by 1.0% and 3.8%, respectively, in 2020 compared with 2019 (Fig. 2D).

**Table 1:** The proportion of changes in the number of newly diagnosed cases of cervical, endometrial, and ovarian cancer in 2020 compared with 2019, 22 SEER registries.

Cancer type	Cancer stage			
	Total	Localized	Regional	Distant
Cervical cancer	−8.7%	−14.8%	−5.9%	+1.0%
Endometrial cancer	−5.5%	−7.6%	−2.4%	+3.8%
Ovarian cancer	−6.5%	−12.6%	−13.4%	−1.7%

**Figure 2.**
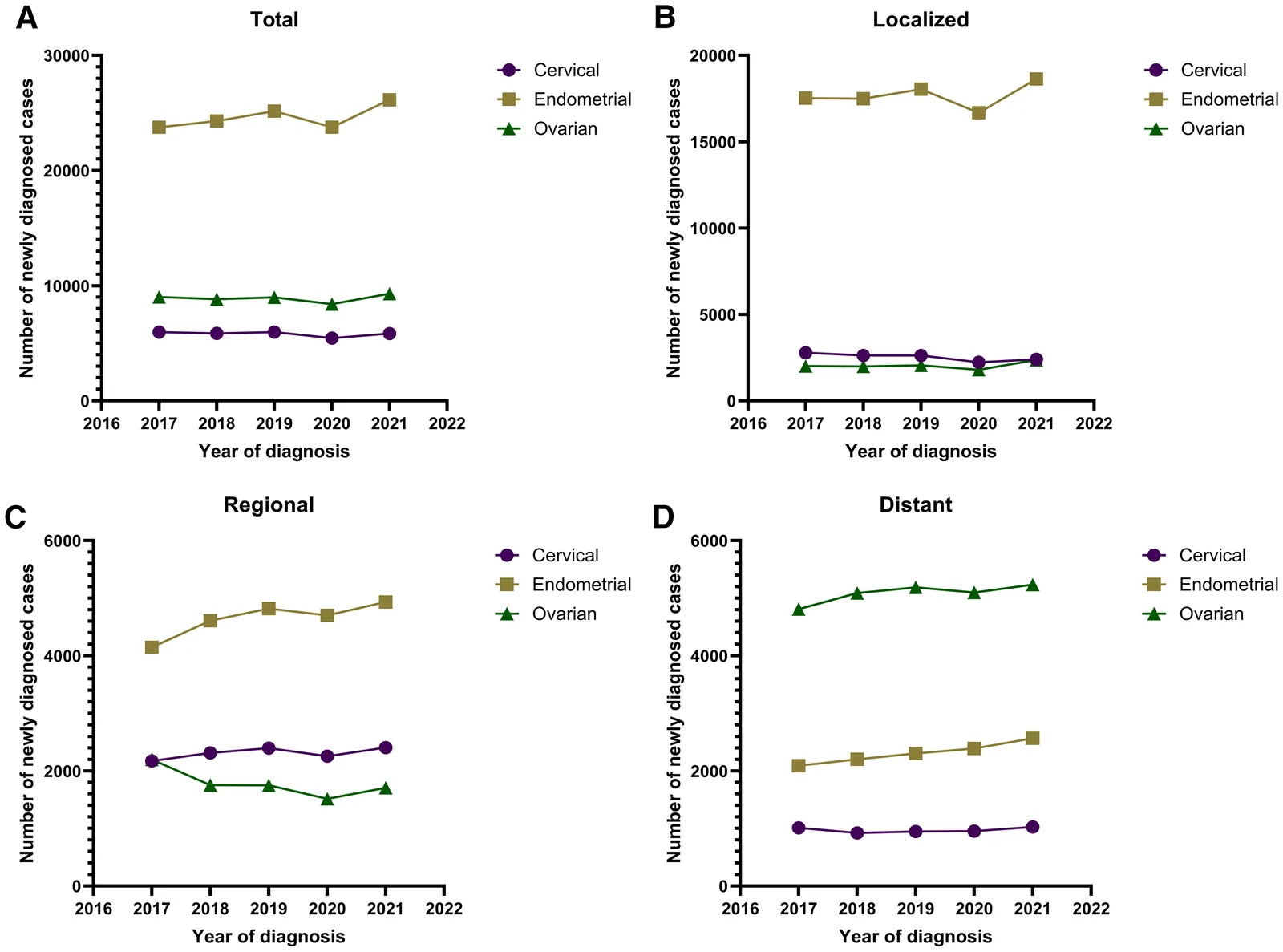
Total number of cases of the three major gynecologic cancers diagnosed in the SEER-22 dataset from 2017 to 2021 (A), and the number of cases categorized by stage as localized (B), regional (C), and distant (D).

### 3.2. Impact of the COVID-19 pandemic on cancer diagnosis

In the SEER-17 dataset, a total of 7997 cervical cancer, 36,151 endometrial cancer, and 11,508 ovarian cancer patients were included. Detailed baseline characteristics are provided in Supplemental Digital Content (Tables S1, S2, and S3, Supplemental Digital Content 3).

Table 2 summarizes the association between diagnostic period and stage at diagnosis. In univariable ordinal logistic regression, patients with cervical cancer (OR, 1.11; 95%CI, 1.02–1.21; *P* = .020) and endometrial cancer (OR, 1.07; 95% CI, 1.01–1.12; *P* = .010) in the pandemic cohort had higher odds of presenting at a more advanced stage than those in the pre-pandemic cohort, whereas no significant association was observed for ovarian cancer (OR, 1.03; 95% CI, 0.96–1.12; *P* = .382). After adjustment for age, race, marital status, median household income, rural-urban continuum, histological type, and tumor grade, the association remained significant for endometrial cancer (adjusted OR, 1.06; 95% CI, 1.01–1.12; *P* = .027) but not for cervical cancer (adjusted OR, 1.07; 95% CI, 0.98–1.18; *P* = .134) or ovarian cancer (adjusted OR, 1.03; 95% CI, 0.94–1.12; *P* = .558). The pre-pandemic cohort served as the reference category. Complete unadjusted and adjusted results, including reference categories for all predictors, are presented in Supplemental Digital Content (Tables S4, S5, and S6, Supplemental Digital Content 4). Model diagnostics supported the use of ordinal models: the proportional-odds assumption was satisfied for the primary explanatory variable (diagnostic period) in all 3 cancer-specific models (Brant test *P* = .961, .586, and .475 for cervical, endometrial, and ovarian cancer, respectively), and all adjusted generalized variance inflation factors were below 1.3, indicating no concerning multicollinearity (Supplementary Table S12, Supplemental Digital Content 5). Sensitivity analyses using multinomial logistic regression yielded conclusions consistent with the ordinal models (Supplementary Table S13, Supplemental Digital Content 6).

**Table 2:** Ordinal logistic regression to determine odds ratios and 95% confidence intervals for advanced-stage cervical, endometrial, and ovarian cancer diagnosed in 2020 (Pandemic cohort) relative to 2018-2019 (Pre-pandemic cohort), 17 SEER registries, 2018-2020.

Cancer type	Univariable^1^		Multivariable^2^	
	OR (95% CI)	*P*–value	OR (95% CI)	*P*–value
Cervical cancer	1.11 (1.02, 1.21)	.020	1.07 (0.98, 1.18)	.134
Endometrial cancer	1.07 (1.01, 1.12)	.010	1.06 (1.01, 1.12)	.027
Ovarian cancer	1.03 (0.96, 1.12)	.382	1.03 (0.94, 1.12)	.558

1 Cancer stage was an ordinal dependent variable (localized ＜ regional ＜ distant), and an ordinal logistic regression model was used to confirm the relationship between year of diagnosis (2020 vs 2018–2019) and stage.

2 The adjusted variables included age, race, marital status, median household income, rural-urban continuum, histology, and grade.

Supplemental Digital Content (Tables S7, S8, and S9, Supplemental Digital Content 7) presents predictors of diagnosis in 2020 (the pandemic cohort) versus 2018-2019 (the pre-pandemic cohort) for the 3 major gynecologic cancers. For cervical and endometrial cancers, patients with a median household income ≥$75,000 were more likely to be diagnosed in 2020 (reference: <$75,000; OR > 1, *P* < .05). Additionally, for cervical and endometrial cancers, patients diagnosed in 2020 were less likely to reside in areas with a population > 250,000 (urban areas) (OR < 1, *P* < .05).

### 3.3. Impact of the COVID-19 pandemic on treatment and prognosis

Table 3 presents the time from diagnosis to treatment initiation. The distributions of time from diagnosis to treatment initiation and follow-up duration departed from normality in all cancer-specific cohorts (Anderson–Darling test, all *P* < .001; Supplementary Figure S1, Supplemental Digital Content 1; Supplementary Table S11, Supplemental Digital Content 2); therefore, these variables are summarized as medians with interquartile ranges and compared between cohorts using Mann–Whitney *U* tests. The median intervals in the pre-pandemic and pandemic cohorts were 30 and 28 days for cervical cancer (*P* = .019), 31 and 28 days for endometrial cancer (*P* < .001), and 4 and 6 days for ovarian cancer (*P* = .127), respectively.

**Table 3:** Time from diagnosis to treatment initiation by diagnostic period, 17 SEER registries, 2018–2020.

Cancer type	Pre-pandemic cohort (2018–2019), median (IQR), d	Pandemic cohort (2020), median (IQR), d	*P*-value
Cervical cancer	30 (0, 53)	28 (0, 51)	.019
Endometrial cancer	31 (10, 49)	28 (10, 44)	<.001
Ovarian cancer	4 (0, 23)	6 (0, 23)	.127

IQR, interquartile range. *P* values were calculated using the Mann–Whitney *U* test. Patients diagnosed in 2018 to 2019 constituted the pre-pandemic cohort, and those diagnosed in 2020 constituted the pandemic cohort. 9 patients whose time from diagnosis to treatment was top-coded as ”731+ days” in SEER were excluded.

Figure 3 demonstrates the impact of the COVID-19 pandemic in 2020 on CSS. Median follow-up durations for the pre-pandemic and pandemic cohorts were 42 and 27 months for cervical cancer, 44 and 28 months for endometrial cancer, and 40 and 27 months for ovarian cancer, respectively. Compared with patients diagnosed in 2018 to 2019 (the pre-pandemic cohort), those diagnosed in 2020 (the pandemic cohort) exhibited borderline worse CSS for cervical cancer (HR, 1.11; 95% CI, 1.00–1.24; *P* = .050; Figure 3A) and significantly worse CSS for endometrial cancer (HR, 1.10; 95% CI, 1.02–1.18; *P* = .009; Figure 3B), whereas no significant difference in CSS was observed for ovarian cancer (HR, 1.05; 95% CI, 0.98–1.14; *P* = .185; Figure 3C). The corresponding 18-month CSS rates for the pre-pandemic and pandemic cohorts were 86.6% and 84.5% for cervical cancer, 93.1% and 92.6% for endometrial cancer, and 82.8% and 83.0% for ovarian cancer, respectively. Supplemental Digital Content Table S10, Supplemental Digital Content 8 provides detailed 6-month, 12-month, and 18-month CSS rates for the 3 cancers.

**Figure 3.**
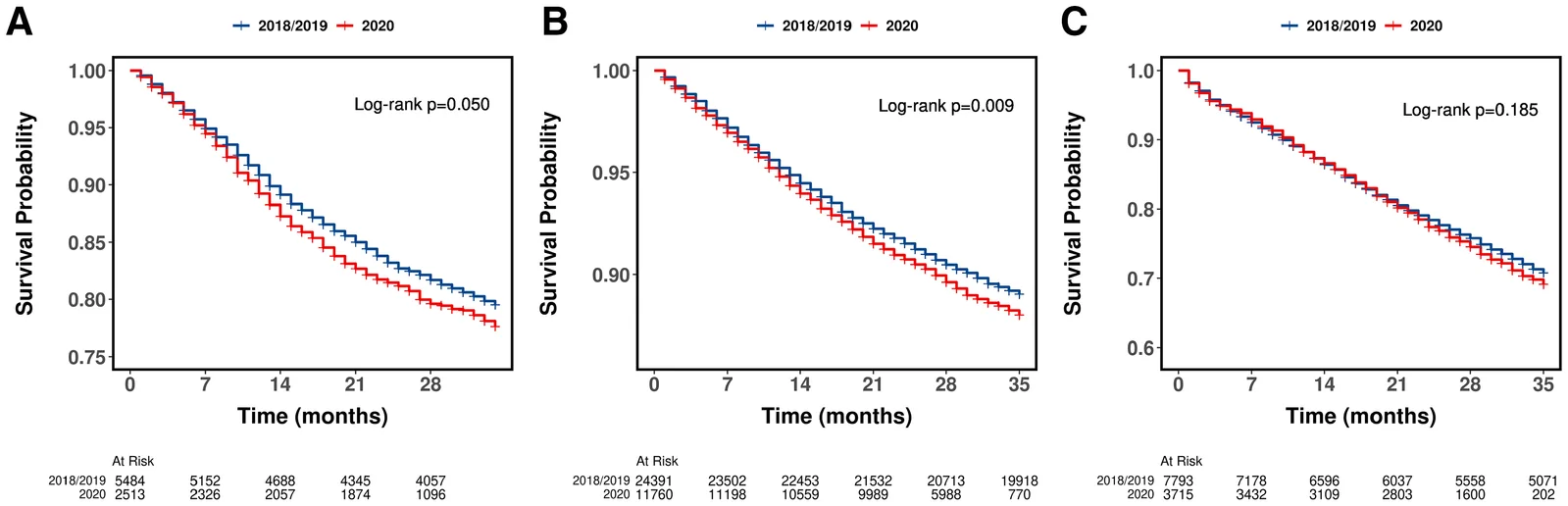
The Kaplan–Meier curves illustrate the cancer-specific survival for cervical cancer (A), endometrial cancer (B), and ovarian cancer (C) diagnosed between 2018 and 2020. Patients diagnosed in 2018 to 2019 constituted the pre-pandemic cohort, and those diagnosed in 2020 constituted the pandemic cohort.

## 4. Discussion

Previous studies have demonstrated that the COVID-19 pandemic in 2020 exerted multidimensional adverse effects on cancer management, encompassing cancer screening, diagnosis, and treatment.^[14–16]^ These negative impacts were particularly pronounced in low- and middle-income countries, as well as in nations implementing stringent lockdown measures.^[14]^ Furthermore, cancer patients during the pandemic experienced increased economic hardship and deterioration in psychosocial well-being.^[17,18]^ Therefore, a comprehensive understanding of these effects is essential for informing future healthcare policies and strengthening support systems to mitigate adverse consequences in similar future crises.

Consistent with prior research, our study observed declines in newly diagnosed cases across all three major gynecologic cancers in 2020 compared with 2019, albeit with varying magnitudes.^[14,19,20]^ A contributing factor may be the widespread reduction in cancer screening during the pandemic.^[15,21]^ For instance, a meta-analysis by Teglia et al reported that colorectal cancer screening rates decreased by 44.9%, while breast and cervical cancer screening rates declined by 40% to 50% between January and October 2020.^[21]^ Multiple studies have documented significant decreases in cervical cancer screening coverage during the pandemic. A multicenter retrospective cohort study from Taiwan reported a 4.5% reduction in total Pap smear examinations, with abnormal smear rates increasing from 2.44% to 3.61% during the pandemic period.^[5]^ Similarly, a systematic review and meta-analysis from Italy found significant reductions in colposcopy services, cervical treatments, and diagnoses of both precancerous lesions and invasive cancers during the pandemic, with more pronounced declines observed in countries with screening coverage below 70%.^[6]^ Additionally, the pandemic may have led to delayed medical consultation among asymptomatic or mildly symptomatic patients, thereby reducing the number of newly diagnosed cases while increasing the proportion of advanced-stage presentations.^[21,22]^ Importantly, the decrease in newly recorded diagnoses should not be interpreted as evidence of a true reduction in the underlying occurrence of gynecologic cancers. Instead, it likely reflects pandemic-related disruption of cancer screening and diagnostic pathways, reduced in-person healthcare utilization, patient concerns regarding severe acute respiratory syndrome coronavirus 2 exposure, and the prioritization of COVID-19-related care during lockdown periods. Patients with mild, nonspecific, or asymptomatic disease may have deferred medical evaluation, whereas those with more severe symptoms continued to seek care. This pattern is consistent with our observation of a relatively greater reduction in localized and regional disease and a smaller reduction or, for cervical and endometrial cancers, an increase in distant-stage diagnoses.

As revealed by our stage-specific analysis, the decline in new cases of distant metastatic disease was markedly smaller than that observed for localized and regional disease. Notably, cases of distant metastatic cervical and endometrial cancers actually increased. These findings were further corroborated by ordinal logistic regression analyses, which demonstrated that cervical and endometrial cancers were diagnosed at more advanced stages in 2020 (the pandemic cohort) compared with 2018-2019 (the pre-pandemic cohort). The absence of this phenomenon in ovarian cancer may be attributable to its insidious onset, with over 70% of patients presenting at an advanced stage at initial diagnosis; the stage distribution is already saturated at presentation, making it difficult for pandemic-related diagnostic delays to further shift the proportion toward advanced disease. Consequently, the stage migration pattern observed in cervical and endometrial cancers was not evident in ovarian cancer.

Although the overall number of diagnoses decreased for all three cancers during the COVID-19 pandemic, the magnitude of reduction appeared to vary across demographic subgroups. For cervical and endometrial cancers, patients with a median household income ≥$75,000 were more likely to be diagnosed in 2020, potentially reflecting that higher socioeconomic status groups maintained better healthcare access during the pandemic. Previous studies have indicated that women with lower socioeconomic status and limited educational attainment face greater barriers to screening,^[23–25]^ and the COVID-19 pandemic may have exacerbated these disparities higher-income individuals were more likely to overcome pandemic-related healthcare restrictions through private health insurance, telemedicine, or flexible appointment scheduling, thereby ensuring continuity of cancer screening and diagnosis. Another noteworthy observation is that, for cervical and endometrial cancers, patients diagnosed in 2020 were less likely to reside in urban areas with a population >250,000. This shift may partially reflect healthcare resource reallocation to address surging COVID-19 cases in major metropolitan areas, resulting in reduced availability of routine services such as cancer screening and diagnosis. Additionally, more stringent lockdown measures and public health restrictions in densely populated areas may have further limited healthcare accessibility and deterred individuals from seeking medical care. These factors collectively undermined the conventional advantages of early detection and screening in urban environments, highlighting the influence of healthcare accessibility and pandemic response strategies on cancer diagnostic patterns.^[22]^

Previous studies have extensively reported treatment delays during the COVID-19 pandemic, with surgical treatment reductions being more pronounced than those for medical therapy.^[14,21,26–28]^ However, in the present study, no delays from diagnosis to treatment were observed for any of the 3 cancers; indeed, time intervals were even shortened for cervical and endometrial cancers. A previous SEER database-based study has noted this phenomenon.^[29]^ On 1 hand, this may be attributable to increased treatment urgency resulting from the higher proportion of advanced-stage patients. On the other hand, treatment delays or reductions during the pandemic exhibited a U-shaped pattern from January to October 2020, with later months showing diminished pandemic impact.^[21]^ However, our study could not distinguish monthly variations, as it provides average comparisons that may not fully capture the most accurate temporal dynamics. Additionally, a retrospective cohort study from Montreal, Canada, similarly observed no delays in diagnosis-to-treatment intervals for cervical cancer during the COVID-19 pandemic.^[30]^

The multidimensional impact of the COVID-19 pandemic on cancer management has raised concerns regarding adverse oncological outcomes. In this study, the pandemic cohort exhibited significantly worse CSS for endometrial cancer and borderline worse CSS for cervical cancer, whereas no clear difference in prognosis was observed for ovarian cancer. The potential explanations for these findings are multifaceted. First, the prognostic deterioration may be closely associated with diagnostic delays and changes in stage distribution. As previously noted, the absolute number of distant-stage diagnoses of cervical and endometrial cancers increased in 2020 despite an overall decline in new cases, and the adjusted ordinal regression confirmed a shift toward more advanced-stage disease for endometrial cancer; advanced-stage presentation is typically accompanied by more limited treatment options and poorer survival outcomes. Second, healthcare resource reallocation during the pandemic may have led to reduced treatment intensity or modified treatment protocols, such as surgical delays, extended chemotherapy intervals, or radiotherapy interruptions factors that may particularly impact patients with cervical and endometrial cancers, who generally have more favorable prognoses. In contrast, ovarian cancer is characterized by an inherently poor prognosis and extremely high rates of advanced-stage presentation; survival outcomes are primarily driven by tumor biological behavior, with pandemic-related healthcare disruptions having relatively limited marginal effects. Furthermore, ovarian cancer patients often require urgent debulking surgery or chemotherapy; the imperative nature of their treatment may have secured relatively prioritized access to healthcare resources during the pandemic, thereby partially offsetting the negative impact of systemic healthcare pressures. A retrospective cohort study from Kaiser Permanente Southern California corroborates our findings, demonstrating no significant difference in 12-month recurrence rates between ovarian cancer patients diagnosed during versus before the pandemic.^[10]^ Notably, the relatively short follow-up of the pandemic cohort (median, 27–28 months across the three cancers) may have limited our ability to detect longer-term survival differences, particularly for ovarian cancer, underscoring the need for extended follow-up to elucidate the pandemic’s impact on cancer patient survival.

This study has several limitations. First, the geographic coverage of the SEER database is limited and may not be representative of the entire U.S. population. Second, the database lacks critical clinical information including comorbidities, specific treatment regimens, and COVID-19 infection status. Third, the annual-level analysis cannot capture monthly dynamic variations in pandemic impact. Fourth, the relatively short follow-up duration limits long-term survival assessment. Fifth, the retrospective design precludes the establishment of causal relationships. Finally, this study focused exclusively on the three major gynecologic cancers, limiting generalizability.

## 5. Conclusion

The COVID-19 pandemic was associated with fewer recorded diagnoses of cervical, endometrial, and ovarian cancers in 2020. This decline should be interpreted cautiously because it may partly reflect reduced screening, delayed presentation, healthcare avoidance, and changes in healthcare-seeking behavior rather than a true reduction in cancer occurrence. Diagnosis during the pandemic was associated with a modest shift toward more advanced-stage endometrial cancer and with worse short-term CSS for cervical and endometrial cancers, whereas no clear association was observed for ovarian cancer. Time from diagnosis to treatment initiation did not increase during the pandemic and was even shorter for cervical and endometrial cancers. Maintaining access to cancer screening, diagnostic services, and timely treatment should remain a priority during future public health emergencies.

## Author contributions

**Conceptualization:** Chunyan Tan, Ning Xu.

**Data curation:** Chunyan Tan, Huibo Dai, Xingya Liu, Ning Xu, Xinli Zhong.

**Formal analysis:** Chunyan Tan, Huibo Dai, Xingya Liu, Ning Xu, Xinli Zhong.

**Methodology:** Chunyan Tan, Huibo Dai, Yanqiu Liu.

**Software:** Chunyan Tan, Xinli Zhong, Yanqiu Liu.

**Validation:** Chunyan Tan.

**Resources:** Huibo Dai.

**Investigation:** Xingya Liu.

**Project administration:** Ning Xu.

**Supervision:** Ning Xu.

**Visualization:** Ning Xu.

**Writing – original draft:** Chunyan Tan, Huibo Dai, Xingya Liu, Ning Xu, Xinli Zhong, Yanqiu Liu.

**Writing – review & editing:** Chunyan Tan, Huibo Dai, Xingya Liu, Ning Xu, Xinli Zhong, Yanqiu Liu.




























