# Supplementary material for: Impact of the COVID-19 pandemic on diagnosis, treatment, and prognosis of cervical, endometrial, and ovarian cancers: A retrospective cohort study based on the SEER database
Source: Medicine (Baltimore). 2026 Oct 16;105(42):e51038. doi: 10.1097/MD.0000000000051038 (PMC13681103; doi:10.1097/MD.0000000000051038)
Supplement: Supplementary file 1 [file medi-105-e51038-s002.docx]

Table S11. Distribution assessment of continuous variables

| Cancer type | Variable | Cohort | N | Anderson–Darling P value | Distribution | Summary used |
| --- | --- | --- | --- | --- | --- | --- |
| Cervical | Time to treatment, days | Pre-pandemic | 5,482 | < .001 | Non-normal | Median (IQR) |
| Cervical | Time to treatment, days | Pandemic | 2,512 | < .001 | Non-normal | Median (IQR) |
| Endometrial | Time to treatment, days | Pre-pandemic | 24,385 | < .001 | Non-normal | Median (IQR) |
| Endometrial | Time to treatment, days | Pandemic | 11,760 | < .001 | Non-normal | Median (IQR) |
| Ovarian | Time to treatment, days | Pre-pandemic | 7,793 | < .001 | Non-normal | Median (IQR) |
| Ovarian | Time to treatment, days | Pandemic | 3,715 | < .001 | Non-normal | Median (IQR) |
| Cervical | Follow-up, months | Pre-pandemic | 5,484 | < .001 | Non-normal | Median (IQR) |
| Cervical | Follow-up, months | Pandemic | 2,513 | < .001 | Non-normal | Median (IQR) |
| Endometrial | Follow-up, months | Pre-pandemic | 24,391 | < .001 | Non-normal | Median (IQR) |
| Endometrial | Follow-up, months | Pandemic | 11,760 | < .001 | Non-normal | Median (IQR) |
| Ovarian | Follow-up, months | Pre-pandemic | 7,793 | < .001 | Non-normal | Median (IQR) |
| Ovarian | Follow-up, months | Pandemic | 3,715 | < .001 | Non-normal | Median (IQR) |

IQR, interquartile range. Normality was assessed using the Anderson–Darling test and supported by histograms and quantile–quantile plots (Supplementary Figure S1).
