# Supplementary material for: Impact of the COVID-19 pandemic on diagnosis, treatment, and prognosis of cervical, endometrial, and ovarian cancers: A retrospective cohort study based on the SEER database
Source: Medicine (Baltimore). 2026 Oct 16;105(42):e51038. doi: 10.1097/MD.0000000000051038 (PMC13681103; doi:10.1097/MD.0000000000051038)
Supplement: Supplementary file 2 [file medi-105-e51038-s003.docx]

Table S1. Baseline characteristics of patients diagnosed with cervical cancer, 17 SEER registries, 2018-2020

| **Characteristic** | **Overall**  **N = 7,997** | **Pre-pandemic cohort (2018–2019)**  **N = 5,484** | **Pandemic cohort (2020)**  **N = 2,513** | **p-value** |
| --- | --- | --- | --- | --- |
| **Age** |  |  |  | .145 |
| 20~49 | 4,226 (52.8%) | 2,924 (53.3%) | 1,302 (51.8%) |  |
| 50~64 | 2,369 (29.6%) | 1,635 (29.8%) | 734 (29.2%) |  |
| 65~74 | 946 (11.8%) | 626 (11.4%) | 320 (12.7%) |  |
| 75~85 | 456 (5.7%) | 299 (5.5%) | 157 (6.2%) |  |
| **Race** |  |  |  | .472 |
| White | 5,952 (74.4%) | 4,087 (74.5%) | 1,865 (74.2%) |  |
| Black | 1,065 (13.3%) | 740 (13.5%) | 325 (12.9%) |  |
| Other | 980 (12.3%) | 657 (12.0%) | 323 (12.9%) |  |
| **Marital status** |  |  |  | .643 |
| Unmarried | 4,441 (55.5%) | 3,055 (55.7%) | 1,386 (55.2%) |  |
| Married | 3,556 (44.5%) | 2,429 (44.3%) | 1,127 (44.8%) |  |
| **Median household income^*^** |  |  |  | .074 |
| ＜75,000USD | 2,309 (28.9%) | 1,617 (29.5%) | 692 (27.5%) |  |
| ≥75,000USD | 5,688 (71.1%) | 3,867 (70.5%) | 1,821 (72.5%) |  |
| **Rural-Urban continuum** |  |  |  | .221 |
| Rural | 919 (11.5%) | 614 (11.2%) | 305 (12.1%) |  |
| Urban | 7,078 (88.5%) | 4,870 (88.8%) | 2,208 (87.9%) |  |
| **Stage** |  |  |  | .061 |
| Localized | 3,457 (43.2%) | 2,412 (44.0%) | 1,045 (41.6%) |  |
| Regional | 3,350 (41.9%) | 2,284 (41.6%) | 1,066 (42.4%) |  |
| Distant | 1,190 (14.9%) | 788 (14.4%) | 402 (16.0%) |  |
| **Histology** |  |  |  | .104 |
| squamous | 5,191 (64.9%) | 3,582 (65.3%) | 1,609 (64.0%) |  |
| adenocarcinoma | 2,209 (27.6%) | 1,511 (27.6%) | 698 (27.8%) |  |
| adenosquamous | 210 (2.6%) | 147 (2.7%) | 63 (2.5%) |  |
| other | 387 (4.8%) | 244 (4.4%) | 143 (5.7%) |  |
| **Grade** |  |  |  | .275 |
| Well differentiated | 986 (12.3%) | 688 (12.5%) | 298 (11.9%) |  |
| Moderately differentiated | 2,682 (33.5%) | 1,863 (34.0%) | 819 (32.6%) |  |
| Poorly differentiated | 2,150 (26.9%) | 1,471 (26.8%) | 679 (27.0%) |  |
| Unknown | 2,179 (27.2%) | 1,462 (26.7%) | 717 (28.5%) |  |
| **Local surgery** |  |  |  | .065 |
| Yes | 4,392 (54.9%) | 3,050 (55.6%) | 1,342 (53.4%) |  |
| None/Unknown | 3,605 (45.1%) | 2,434 (44.4%) | 1,171 (46.6%) |  |
| **Radiotherapy** |  |  |  | .328 |
| Yes | 4,879 (61.0%) | 3,326 (60.6%) | 1,553 (61.8%) |  |
| None/Unknown | 3,118 (39.0%) | 2,158 (39.4%) | 960 (38.2%) |  |
| **Chemotherapy** |  |  |  | .050 |
| Yes | 4,703 (58.8%) | 3,185 (58.1%) | 1,518 (60.4%) |  |
| No/Unknown | 3,294 (41.2%) | 2,299 (41.9%) | 995 (39.6%) |  |
| **Follow-up months, median (IQR)** | 34 (24, 46) | 42 (26, 50) | 27 (20, 31) | <.001 |

USD, United States dollar; IQR, interquartile range. ^*^Adjusted to 2023.
