# Supplementary material for: Impact of the COVID-19 pandemic on diagnosis, treatment, and prognosis of cervical, endometrial, and ovarian cancers: A retrospective cohort study based on the SEER database
Source: Medicine (Baltimore). 2026 Oct 16;105(42):e51038. doi: 10.1097/MD.0000000000051038 (PMC13681103; doi:10.1097/MD.0000000000051038)
Supplement: Supplementary file 3 [file medi-105-e51038-s004.docx]

Table S4. Ordered logistic regression identifying predictors of advanced stage in cervical cancer, 17 SEER registries, 2018-2020

| **Characteristic** | **Univariable** | | **Multivariable** | |
| --- | --- | --- | --- | --- |
|  | **OR (95% CI)** | **p-value** | **OR (95% CI)** | **p-value** |
| **Year of diagnosis** |  |  |  |  |
| 2018-2019 | Ref |  | Ref |  |
| 2020 | 1.11 (1.02, 1.21) | .020 | 1.07 (0.98, 1.18) | .134 |
| **Age** |  |  |  |  |
| 20~49 | Ref |  | Ref |  |
| 50~64 | 2.15 (1.95, 2.37) | <.001 | 2.02 (1.83, 2.24) | <.001 |
| 65~74 | 2.95 (2.58, 3.37) | <.001 | 2.55 (2.22, 2.92) | <.001 |
| 75~85 | 3.65 (3.05, 4.37) | <.001 | 3.11 (2.59, 3.74) | <.001 |
| **Race** |  |  |  |  |
| White | Ref |  | Ref |  |
| Black | 1.40 (1.24, 1.58) | <.001 | 1.15 (1.01, 1.31) | .035 |
| Other | 1.01 (0.89, 1.15) | .856 | 0.99 (0.86, 1.13) | .841 |
| **Marital status** |  |  |  |  |
| Unmarried | Ref |  | Ref |  |
| Married | 0.71 (0.65, 0.77) | <.001 | 0.77 (0.71, 0.84) | <.001 |
| **Median household income^*^** |  |  |  |  |
| ＜75,000USD | Ref |  | Ref |  |
| ≥75,000USD | 0.85 (0.77, 0.93) | <.001 | 0.93 (0.84, 1.04) | .225 |
| **Rural-Urban continuum** |  |  |  |  |
| Rural | Ref |  | Ref |  |
| Urban | 0.84 (0.74, 0.95) | .006 | 0.94 (0.80, 1.09) | .385 |
| **Histology** |  |  |  |  |
| squamous | Ref |  | Ref |  |
| adenocarcinoma | 0.51 (0.46, 0.56) | <.001 | 0.67 (0.61, 0.75) | <.001 |
| adenosquamous | 0.87 (0.67, 1.12) | .279 | 0.82 (0.63, 1.07) | .152 |
| other | 1.98 (1.62, 2.42) | <.001 | 1.35 (1.10, 1.67) | .005 |
| **Grade** |  |  |  |  |
| Well differentiated | Ref |  | Ref |  |
| Moderately differentiated | 3.78 (3.20, 4.48) | <.001 | 3.14 (2.64, 3.75) | <.001 |
| Poorly differentiated | 7.85 (6.62, 9.34) | <.001 | 5.97 (5.00, 7.16) | <.001 |
| Unknown | 8.01 (6.75, 9.54) | <.001 | 6.31 (5.29, 7.55) | <.001 |

Abbreviation: USD, United States dollar

^*^Adjusted to 2023
