# Supplementary material for: Impact of the COVID-19 pandemic on diagnosis, treatment, and prognosis of cervical, endometrial, and ovarian cancers: A retrospective cohort study based on the SEER database
Source: Medicine (Baltimore). 2026 Oct 16;105(42):e51038. doi: 10.1097/MD.0000000000051038 (PMC13681103; doi:10.1097/MD.0000000000051038)
Supplement: Supplementary file 4 [file medi-105-e51038-s005.docx]

Table S12. Regression model diagnostics

| Cancer type | Brant test, omnibus P value | Brant test P value, diagnostic period | Proportional odds for primary variable | Maximum GVIF (adjusted GVIF) | Multicollinearity | Final model |
| --- | --- | --- | --- | --- | --- | --- |
| Cervical | < .001 | .961 | Satisfied | 1.33 (1.15) | No concerning multicollinearity | Ordinal + multinomial sensitivity |
| Endometrial | < .001 | .586 | Satisfied | 1.87 (1.16) | No concerning multicollinearity | Ordinal + multinomial sensitivity |
| Ovarian | < .001 | .475 | Satisfied | 3.10 (1.20) | No concerning multicollinearity | Ordinal + multinomial sensitivity |

The proportional-odds assumption was assessed using the Brant test. Although the omnibus test was statistically significant in all three models—reflecting the high sensitivity of the test to trivial deviations in large samples (N = 7,997–36,151)—the assumption was satisfied for the primary explanatory variable (diagnostic period) in all cancer-specific models. Multicollinearity was assessed using generalized variance inflation factors (GVIFs); adjusted GVIFs (GVIF^(1/(2×df))) were all below 1.3. Robustness was verified using multinomial logistic regression (Supplementary Table S13).
