# Supplementary material for: Impact of the COVID-19 pandemic on diagnosis, treatment, and prognosis of cervical, endometrial, and ovarian cancers: A retrospective cohort study based on the SEER database
Source: Medicine (Baltimore). 2026 Oct 16;105(42):e51038. doi: 10.1097/MD.0000000000051038 (PMC13681103; doi:10.1097/MD.0000000000051038)
Supplement: Supplementary file 5 [file medi-105-e51038-s006.docx]

Table S13. Multinomial logistic regression sensitivity analysis for stage at diagnosis

| Cancer type | Comparison | OR (95% CI) | P value |
| --- | --- | --- | --- |
| Cervical | Regional vs Localized | 1.07 (0.96, 1.19) | .244 |
| Cervical | Distant vs Localized | 1.13 (0.97, 1.31) | .119 |
| Endometrial | Regional vs Localized | 1.04 (0.98, 1.10) | .199 |
| Endometrial | Distant vs Localized | 1.09 (1.00, 1.20) | .049 |
| Ovarian | Regional vs Localized | 0.97 (0.85, 1.10) | .590 |
| Ovarian | Distant vs Localized | 1.01 (0.90, 1.14) | .872 |

Multivariable multinomial logistic regression with localized stage as the reference outcome, adjusted for age, race, marital status, median household income, rural-urban continuum, histological type, and tumor grade. ORs represent the association of diagnosis in 2020 (pandemic cohort) versus 2018–2019 (pre-pandemic cohort). Results were consistent with the ordinal logistic regression models.
