# Supplementary material for: Impact of the COVID-19 pandemic on diagnosis, treatment, and prognosis of cervical, endometrial, and ovarian cancers: A retrospective cohort study based on the SEER database
Source: Medicine (Baltimore). 2026 Oct 16;105(42):e51038. doi: 10.1097/MD.0000000000051038 (PMC13681103; doi:10.1097/MD.0000000000051038)
Supplement: Supplementary file 6 [file medi-105-e51038-s007.docx]

Table S7. Predictors of cervical cancer diagnosis in 2020 (Pandemic cohort) (compared to 2018-2019 [Pre-pandemic cohort]), 17 SEER registries, 2018-2020

| **Characteristic** | **Univariable** | | **Multivariable** | |
| --- | --- | --- | --- | --- |
|  | **OR (95% CI)** | **p-value** | **OR (95% CI)** | **p-value** |
| **Age** |  |  |  |  |
| 20~49 | Ref |  | Ref |  |
| 50~64 | 1.01 (0.90, 1.12) | .883 | 0.98 (0.88, 1.10) | .747 |
| 65~74 | 1.15 (0.99, 1.33) | .071 | 1.10 (0.94, 1.28) | .231 |
| 75~85 | 1.18 (0.96, 1.45) | .113 | 1.13 (0.92, 1.39) | .243 |
| **Race** |  |  |  |  |
| White | Ref |  | Ref |  |
| Black | 0.96 (0.84, 1.11) | .596 | 0.98 (0.85, 1.13) | .755 |
| Other | 1.08 (0.93, 1.24) | .311 | 1.06 (0.91, 1.22) | .466 |
| **Marital status** |  |  |  |  |
| Unmarried | Ref |  | Ref |  |
| Married | 1.02 (0.93, 1.12) | .643 | 1.02 (0.93, 1.12) | .689 |
| **Median household income^*^** |  |  |  |  |
| ＜75,000USD | Ref |  | Ref |  |
| ≥75,000USD | 1.10 (0.99, 1.22) | .074 | 1.16 (1.03, 1.31) | .016 |
| **Rural-Urban continuum** |  |  |  |  |
| Rural | Ref |  | Ref |  |
| Urban | 0.91 (0.79, 1.06) | .221 | 0.82 (0.70, 0.97) | .022 |
| **Stage** |  |  |  |  |
| Localized | Ref |  | Ref |  |
| Regional | 1.08 (0.97, 1.19) | .156 | 1.07 (0.96, 1.19) | .249 |
| Distant | 1.18 (1.02, 1.35) | .023 | 1.13 (0.98, 1.32) | .102 |
| **Histology** |  |  |  |  |
| squamous | Ref |  | Ref |  |
| adenocarcinoma | 1.03 (0.92, 1.14) | .609 | 1.04 (0.93, 1.16) | .515 |
| adenosquamous | 0.95 (0.71, 1.29) | .760 | 0.95 (0.70, 1.28) | .715 |
| other | 1.30 (1.05, 1.62) | .015 | 1.24 (1.00, 1.55) | .053 |
| **Grade** |  |  |  |  |
| Well differentiated | Ref |  | Ref |  |
| Moderately differentiated | 1.01 (0.87, 1.19) | .855 | 1.01 (0.86, 1.19) | .900 |
| Poorly differentiated | 1.07 (0.90, 1.26) | .446 | 1.02 (0.86, 1.22) | .822 |
| Unknown | 1.13 (0.96, 1.33) | .134 | 1.08 (0.91, 1.28) | .395 |

Abbreviation: USD, United States dollar

^*^Adjusted to 2023
