# Supplementary material for: Impact of the COVID-19 pandemic on diagnosis, treatment, and prognosis of cervical, endometrial, and ovarian cancers: A retrospective cohort study based on the SEER database
Source: Medicine (Baltimore). 2026 Oct 16;105(42):e51038. doi: 10.1097/MD.0000000000051038 (PMC13681103; doi:10.1097/MD.0000000000051038)
Supplement: Supplementary file 7 [file medi-105-e51038-s008.docx]

Table S10. 6, 12-, and 18-month cancer-specific survival rates in patients with cervical, endometrial, and ovarian cancer, 17 SEER registries, 2018-2020

| Cancer type | Survival rate (95% CI, %) | | | p-value^*^ |
| --- | --- | --- | --- | --- |
|  | 6-months | 12-months | 18-months |  |
| Cervical cancer |  |  |  | .050 |
| Pre-pandemic cohort (2018–2019) | 95.7 (95.2, 96.3) | 90.8 (90.1, 91.6) | 86.6 (85.6, 87.5) |  |
| Pandemic cohort (2020) | 95.2 (94.4, 96.1) | 89.2 (88.0, 90.5) | 84.5 (83.1, 86.0) |  |
| Endometrial cancer |  |  |  | .009 |
| Pre-pandemic cohort (2018–2019) | 97.7 (97.5, 97.8) | 95.2 (94.9, 95.5) | 93.1 (92.8, 93.4) |  |
| Pandemic cohort (2020) | 97.3 (97.0, 97.6) | 94.8 (94.4, 95.2) | 92.6 (92.1, 93.1) |  |
| Ovarian cancer |  |  |  | .185 |
| Pre-pandemic cohort (2018–2019) | 93.3 (92.8, 93.9) | 88.2 (87.5, 88.9) | 82.8 (82.0, 83.7) |  |
| Pandemic cohort (2020) | 93.8 (93.1, 94.6) | 88.2 (87.2, 89.2) | 83.0 (81.8, 84.2) |  |

**^*^**Log-rank test
