# Supplementary material for: Impact of the COVID-19 pandemic on diagnosis, treatment, and prognosis of cervical, endometrial, and ovarian cancers: A retrospective cohort study based on the SEER database
Source: Medicine (Baltimore). 2026 Oct 16;105(42):e51038. doi: 10.1097/MD.0000000000051038 (PMC13681103; doi:10.1097/MD.0000000000051038)
Supplement: Supplementary file 8 [file medi-105-e51038-s009.docx]

Table S2. Baseline characteristics of patients diagnosed with endometrial cancer, 17 SEER registries, 2018-2020

| **Characteristic** | **Overall**  **N = 36,151** | **Pre-pandemic cohort (2018–2019)**  **N = 24,391** | **Pandemic cohort (2020)**  **N = 11,760** | **p-value** |
| --- | --- | --- | --- | --- |
| **Age** |  |  |  | .005 |
| 20~49 | 4,399 (12.2%) | 2,995 (12.3%) | 1,404 (11.9%) |  |
| 50~64 | 15,669 (43.3%) | 10,673 (43.8%) | 4,996 (42.5%) |  |
| 65~74 | 11,467 (31.7%) | 7,702 (31.6%) | 3,765 (32.0%) |  |
| 75~85 | 4,616 (12.8%) | 3,021 (12.4%) | 1,595 (13.6%) |  |
| **Race** |  |  |  | .167 |
| White | 28,139 (77.8%) | 19,034 (78.0%) | 9,105 (77.4%) |  |
| Black | 3,864 (10.7%) | 2,612 (10.7%) | 1,252 (10.6%) |  |
| Other | 4,148 (11.5%) | 2,745 (11.3%) | 1,403 (11.9%) |  |
| **Marital status** |  |  |  | .591 |
| Unmarried | 16,671 (46.1%) | 11,224 (46.0%) | 5,447 (46.3%) |  |
| Married | 19,480 (53.9%) | 13,167 (54.0%) | 6,313 (53.7%) |  |
| **Median household income^*^** |  |  |  | .034 |
| ＜75,000USD | 8,569 (23.7%) | 5,862 (24.0%) | 2,707 (23.0%) |  |
| ≥75,000USD | 27,582 (76.3%) | 18,529 (76.0%) | 9,053 (77.0%) |  |
| **Rural-Urban continuum** |  |  |  | .232 |
| Rural | 3,761 (10.4%) | 2,505 (10.3%) | 1,256 (10.7%) |  |
| Urban | 32,390 (89.6%) | 21,886 (89.7%) | 10,504 (89.3%) |  |
| **Stage** |  |  |  | .026 |
| Localized | 25,984 (71.9%) | 17,628 (72.3%) | 8,356 (71.1%) |  |
| Regional | 7,242 (20.0%) | 4,843 (19.9%) | 2,399 (20.4%) |  |
| Distant | 2,925 (8.1%) | 1,920 (7.9%) | 1,005 (8.5%) |  |
| **Histology** |  |  |  | .079 |
| Endometrioid | 26,895 (74.4%) | 18,153 (74.4%) | 8,742 (74.3%) |  |
| Serous | 3,412 (9.4%) | 2,282 (9.4%) | 1,130 (9.6%) |  |
| Clear cell | 521 (1.4%) | 328 (1.3%) | 193 (1.6%) |  |
| Carcinosarcoma & Mixed | 3,081 (8.5%) | 2,077 (8.5%) | 1,004 (8.5%) |  |
| Others/NOS | 2,242 (6.2%) | 1,551 (6.4%) | 691 (5.9%) |  |
| **Grade** |  |  |  | .236 |
| Well differentiated | 16,793 (46.5%) | 11,382 (46.7%) | 5,411 (46.0%) |  |
| Moderately differentiated | 8,379 (23.2%) | 5,685 (23.3%) | 2,694 (22.9%) |  |
| Poorly differentiated | 7,403 (20.5%) | 4,945 (20.3%) | 2,458 (20.9%) |  |
| Unknown | 3,576 (9.9%) | 2,379 (9.8%) | 1,197 (10.2%) |  |
| **Local surgery** |  |  |  | <.001 |
| Yes | 34,639 (95.8%) | 23,444 (96.1%) | 11,195 (95.2%) |  |
| None/Unknown | 1,512 (4.2%) | 947 (3.9%) | 565 (4.8%) |  |
| **Radiotherapy** |  |  |  | .227 |
| Yes | 11,178 (30.9%) | 7,492 (30.7%) | 3,686 (31.3%) |  |
| None/Unknown | 24,973 (69.1%) | 16,899 (69.3%) | 8,074 (68.7%) |  |
| **Chemotherapy** |  |  |  | <.001 |
| Yes | 9,317 (25.8%) | 6,107 (25.0%) | 3,210 (27.3%) |  |
| None/Unknown | 26,834 (74.2%) | 18,284 (75.0%) | 8,550 (72.7%) |  |
| **Follow-up months, median (IQR)** | 38 (27, 48) | 44 (37, 52) | 28 (25, 31) | <.001 |

USD, United States dollar; IQR, interquartile range. ^*^Adjusted to 2023.
