# Supplementary material for: Impact of the COVID-19 pandemic on diagnosis, treatment, and prognosis of cervical, endometrial, and ovarian cancers: A retrospective cohort study based on the SEER database
Source: Medicine (Baltimore). 2026 Oct 16;105(42):e51038. doi: 10.1097/MD.0000000000051038 (PMC13681103; doi:10.1097/MD.0000000000051038)
Supplement: Supplementary file 9 [file medi-105-e51038-s010.docx]

Table S3. Baseline characteristics of patients diagnosed with ovarian cancer, 17 SEER registries, 2018–2020

| **Characteristic** | **Overall**  **N = 11,508** | **Pre-pandemic cohort (2018–2019)**  **N = 7,793** | **Pandemic cohort (2020)**  **N = 3,715** | **p-value** |
| --- | --- | --- | --- | --- |
| **Age** |  |  |  | .874 |
| 20~49 | 2,420 (21.0%) | 1,652 (21.2%) | 768 (20.7%) |  |
| 50~64 | 4,419 (38.4%) | 2,978 (38.2%) | 1,441 (38.8%) |  |
| 65~74 | 2,933 (25.5%) | 1,981 (25.4%) | 952 (25.6%) |  |
| 75~85 | 1,736 (15.1%) | 1,182 (15.2%) | 554 (14.9%) |  |
| **Race** |  |  |  | .253 |
| White | 9,075 (78.9%) | 6,171 (79.2%) | 2,904 (78.2%) |  |
| Black | 976 (8.5%) | 663 (8.5%) | 313 (8.4%) |  |
| Other | 1,457 (12.7%) | 959 (12.3%) | 498 (13.4%) |  |
| **Marital status** |  |  |  | .291 |
| Unmarried | 5,336 (46.4%) | 3,587 (46.0%) | 1,749 (47.1%) |  |
| Married | 6,172 (53.6%) | 4,206 (54.0%) | 1,966 (52.9%) |  |
| **Median household income^*^** |  |  |  | .522 |
| ＜75,000USD | 2,647 (23.0%) | 1,806 (23.2%) | 841 (22.6%) |  |
| ≥75,000USD | 8,861 (77.0%) | 5,987 (76.8%) | 2,874 (77.4%) |  |
| **Rural-Urban continuum** |  |  |  | .427 |
| Rural | 1,067 (9.3%) | 711 (9.1%) | 356 (9.6%) |  |
| Urban | 10,441 (90.7%) | 7,082 (90.9%) | 3,359 (90.4%) |  |
| **Stage** |  |  |  | .435 |
| Localized | 2,651 (23.0%) | 1,799 (23.1%) | 852 (22.9%) |  |
| Regional | 2,166 (18.8%) | 1,490 (19.1%) | 676 (18.2%) |  |
| Distant | 6,691 (58.1%) | 4,504 (57.8%) | 2,187 (58.9%) |  |
| **Histology** |  |  |  | .857 |
| High-grade serous carcinoma | 5,448 (47.3%) | 3,671 (47.1%) | 1,777 (47.8%) |  |
| Low-grade serous carcinoma | 371 (3.2%) | 245 (3.1%) | 126 (3.4%) |  |
| Endometrioid carcinoma | 1,351 (11.7%) | 918 (11.8%) | 433 (11.7%) |  |
| Clear cell carcinoma | 938 (8.2%) | 645 (8.3%) | 293 (7.9%) |  |
| Mucinous carcinoma | 705 (6.1%) | 472 (6.1%) | 233 (6.3%) |  |
| Others/NOS | 2,695 (23.4%) | 1,842 (23.6%) | 853 (23.0%) |  |
| **Grade** |  |  |  | .333 |
| Well differentiated | 1,488 (12.9%) | 996 (12.8%) | 492 (13.2%) |  |
| Moderately differentiated | 960 (8.3%) | 663 (8.5%) | 297 (8.0%) |  |
| Poorly differentiated | 5,071 (44.1%) | 3,466 (44.5%) | 1,605 (43.2%) |  |
| Unknown | 3,989 (34.7%) | 2,668 (34.2%) | 1,321 (35.6%) |  |
| **Local surgery** |  |  |  | .204 |
| Yes | 10,083 (87.6%) | 6,849 (87.9%) | 3,234 (87.1%) |  |
| None/Unknown | 1,425 (12.4%) | 944 (12.1%) | 481 (12.9%) |  |
| **Radiotherapy** |  |  |  | .243 |
| Yes | 220 (1.9%) | 157 (2.0%) | 63 (1.7%) |  |
| None/Unknown | 11,288 (98.1%) | 7,636 (98.0%) | 3,652 (98.3%) |  |
| **Chemotherapy** |  |  |  | .629 |
| Yes | 9,190 (79.9%) | 6,233 (80.0%) | 2,957 (79.6%) |  |
| None/Unknown | 2,318 (20.1%) | 1,560 (20.0%) | 758 (20.4%) |  |
| **Follow-up months, median (IQR)** | 33 (22, 44) | 40 (23, 49) | 27 (21, 31) | <.001 |

USD, United States dollar; IQR, interquartile range. *Adjusted to 2023.
