# Supplementary material for: Impact of the COVID-19 pandemic on diagnosis, treatment, and prognosis of cervical, endometrial, and ovarian cancers: A retrospective cohort study based on the SEER database
Source: Medicine (Baltimore). 2026 Oct 16;105(42):e51038. doi: 10.1097/MD.0000000000051038 (PMC13681103; doi:10.1097/MD.0000000000051038)
Supplement: Supplementary file 10 [file medi-105-e51038-s011.docx]

Table S5. Ordered logistic regression identifying predictors of advanced stage in endometrial cancer, 17 SEER registries, 2018-2020

| **Characteristic** | **Univariable** | | **Multivariable** | |
| --- | --- | --- | --- | --- |
|  | **OR (95% CI)** | **p-value** | **OR (95% CI)** | **p-value** |
| **Year of diagnosis** |  |  |  |  |
| 2018-2019 | Ref |  | Ref |  |
| 2020 | 1.07 (1.01, 1.12) | .010 | 1.06 (1.01, 1.12) | .027 |
| **Age** |  |  |  |  |
| 20~49 | Ref |  | Ref |  |
| 50~64 | 1.30 (1.20, 1.40) | <.001 | 1.01 (0.93, 1.10) | .856 |
| 65~74 | 1.55 (1.42, 1.68) | <.001 | 0.90 (0.82, 0.99) | .023 |
| 75~85 | 1.86 (1.70, 2.05) | <.001 | 0.93 (0.84, 1.03) | .159 |
| **Race** |  |  |  |  |
| White | Ref |  | Ref |  |
| Black | 1.80 (1.68, 1.93) | <.001 | 1.01 (0.94, 1.09) | .783 |
| Other | 1.22 (1.14, 1.31) | <.001 | 1.20 (1.11, 1.29) | <.001 |
| **Marital status** |  |  |  |  |
| Unmarried | Ref |  | Ref |  |
| Married | 0.77 (0.73, 0.80) | <.001 | 0.79 (0.75, 0.83) | <.001 |
| **Median household income^*^** |  |  |  |  |
| ＜75,000USD | Ref |  | Ref |  |
| ≥75,000USD | 1.03 (0.97, 1.08) | .338 | 1.02 (0.95, 1.09) | .662 |
| **Rural-Urban continuum** |  |  |  |  |
| Rural | Ref |  | Ref |  |
| Urban | 1.08 (1.00, 1.16) | .053 | 1.01 (0.92, 1.11) | .839 |
| **Histology** |  |  |  |  |
| Endometrioid | Ref |  | Ref |  |
| Serous | 5.72 (5.33, 6.14) | <.001 | 1.81 (1.66, 1.97) | <.001 |
| Clear cell | 4.55 (3.86, 5.37) | <.001 | 1.44 (1.21, 1.71) | <.001 |
| Carcinosarcoma & Mixed | 4.01 (3.73, 4.32) | <.001 | 1.60 (1.47, 1.75) | <.001 |
| Others/NOS | 4.10 (3.76, 4.48) | <.001 | 2.20 (1.99, 2.42) | <.001 |
| **Grade** |  |  |  |  |
| Well differentiated | Ref |  | Ref |  |
| Moderately differentiated | 2.82 (2.64, 3.01) | <.001 | 2.83 (2.64, 3.03) | <.001 |
| Poorly differentiated | 8.68 (8.14, 9.26) | <.001 | 6.72 (6.25, 7.24) | <.001 |
| Unknown | 12.08 (11.15, 13.08) | <.001 | 7.89 (7.17, 8.67) | <.001 |

Abbreviation: USD, United States dollar

^*^Adjusted to 2023
