# Supplementary material for: Impact of the COVID-19 pandemic on diagnosis, treatment, and prognosis of cervical, endometrial, and ovarian cancers: A retrospective cohort study based on the SEER database
Source: Medicine (Baltimore). 2026 Oct 16;105(42):e51038. doi: 10.1097/MD.0000000000051038 (PMC13681103; doi:10.1097/MD.0000000000051038)
Supplement: Supplementary file 11 [file medi-105-e51038-s012.docx]

Table S6. Ordered logistic regression identifying predictors of advanced stage in ovarian cancer, 17 SEER registries, 2018-2020

| **Characteristic** | **Univariable** | | **Multivariable** | |
| --- | --- | --- | --- | --- |
|  | **OR (95% CI)** | **p-value** | **OR (95% CI)** | **p-value** |
| **Year of diagnosis** |  |  |  |  |
| 2018-2019 | Ref |  | Ref |  |
| 2020 | 1.03 (0.96, 1.12) | .382 | 1.03 (0.94, 1.12) | .558 |
| **Age** |  |  |  |  |
| 20~49 | Ref |  | Ref |  |
| 50~64 | 1.94 (1.77, 2.13) | <.001 | 1.48 (1.33, 1.65) | <.001 |
| 65~74 | 3.39 (3.04, 3.77) | <.001 | 2.00 (1.77, 2.26) | <.001 |
| 75~85 | 4.51 (3.97, 5.14) | <.001 | 2.14 (1.85, 2.47) | <.001 |
| **Race** |  |  |  |  |
| White | Ref |  | Ref |  |
| Black | 1.07 (0.93, 1.22) | .349 | 0.94 (0.81, 1.09) | .431 |
| Other | 0.76 (0.68, 0.84) | <.001 | 1.13 (1.00, 1.28) | .049 |
| **Marital status** |  |  |  |  |
| Unmarried | Ref |  | Ref |  |
| Married | 0.98 (0.91, 1.05) | .515 | 0.96 (0.89, 1.05) | .386 |
| **Median household income^*^** |  |  |  |  |
| ＜75,000USD | Ref |  | Ref |  |
| ≥75,000USD | 0.95 (0.87, 1.03) | .235 | 1.14 (1.02, 1.27) | .022 |
| **Rural-Urban continuum** |  |  |  |  |
| Rural | Ref |  | Ref |  |
| Urban | 0.87 (0.77, 0.99) | .037 | 0.84 (0.72, 0.99) | .038 |
| **Histology** |  |  |  |  |
| High-grade serous carcinoma | Ref |  | Ref |  |
| Low-grade serous carcinoma | 0.45 (0.36, 0.57) | <.001 | 2.20 (1.67, 2.91) | <.001 |
| Endometrioid carcinoma | 0.05 (0.05, 0.06) | <.001 | 0.15 (0.12, 0.17) | <.001 |
| Clear cell carcinoma | 0.07 (0.07, 0.09) | <.001 | 0.07 (0.06, 0.08) | <.001 |
| Mucinous carcinoma | 0.04 (0.04, 0.05) | <.001 | 0.10 (0.08, 0.12) | <.001 |
| Others/NOS | 0.23 (0.21, 0.26) | <.001 | 0.26 (0.23, 0.29) | <.001 |
| **Grade** |  |  |  |  |
| Well differentiated | Ref |  | Ref |  |
| Moderately differentiated | 0.85 (0.73, 0.99) | .036 | 1.62 (1.36, 1.92) | <.001 |
| Poorly differentiated | 6.68 (5.96, 7.49) | <.001 | 3.73 (3.17, 4.40) | <.001 |
| Unknown | 5.94 (5.28, 6.68) | <.001 | 5.83 (4.94, 6.90) | <.001 |

Abbreviation: USD, United States dollar

^*^Adjusted to 2023
