# Supplementary material for: Impact of the COVID-19 pandemic on diagnosis, treatment, and prognosis of cervical, endometrial, and ovarian cancers: A retrospective cohort study based on the SEER database
Source: Medicine (Baltimore). 2026 Oct 16;105(42):e51038. doi: 10.1097/MD.0000000000051038 (PMC13681103; doi:10.1097/MD.0000000000051038)
Supplement: Supplementary file 12 [file medi-105-e51038-s013.docx]

Table S8. Predictors of endometrial cancer diagnosis in 2020 (Pandemic cohort) (compared to 2018-2019 [Pre-pandemic cohort]), 17 SEER registries, 2018-2020

| **Characteristic** | **Univariable** | | **Multivariable** | |
| --- | --- | --- | --- | --- |
|  | **OR (95% CI)** | **p-value** | **OR (95% CI)** | **p-value** |
| **Age** |  |  |  |  |
| 20~49 | Ref |  | Ref |  |
| 50~64 | 1.00 (0.93, 1.07) | .968 | 1.00 (0.93, 1.07) | .969 |
| 65~74 | 1.04 (0.97, 1.12) | .270 | 1.04 (0.97, 1.13) | .276 |
| 75~85 | 1.13 (1.03, 1.23) | .008 | 1.12 (1.02, 1.23) | .013 |
| **Race** |  |  |  |  |
| White | Ref |  | Ref |  |
| Black | 1.00 (0.93, 1.08) | .956 | 1.01 (0.94, 1.09) | .841 |
| Other | 1.07 (1.00, 1.14) | .060 | 1.07 (1.00, 1.15) | .060 |
| **Marital status** |  |  |  |  |
| Unmarried | Ref |  | Ref |  |
| Married | 0.99 (0.95, 1.03) | .591 | 0.99 (0.95, 1.04) | .782 |
| **Median household income^*^** |  |  |  |  |
| ＜75,000USD | Ref |  | Ref |  |
| ≥75,000USD | 1.06 (1.00, 1.11) | .034 | 1.09 (1.03, 1.16) | .006 |
| **Rural-Urban continuum** |  |  |  |  |
| Rural | Ref |  | Ref |  |
| Urban | 0.96 (0.89, 1.03) | .232 | 0.90 (0.83, 0.98) | .012 |
| **Stage** |  |  |  |  |
| Localized | Ref |  | Ref |  |
| Regional | 1.05 (0.99, 1.10) | .120 | 1.04 (0.98, 1.10) | .201 |
| Distant | 1.10 (1.02, 1.20) | .016 | 1.10 (1.01, 1.21) | .028 |
| **Histology** |  |  |  |  |
| Endometrioid | Ref |  | Ref |  |
| Serous | 1.03 (0.95, 1.11) | .471 | 0.95 (0.86, 1.04) | .285 |
| Clear cell | 1.22 (1.02, 1.46) | .029 | 1.13 (0.94, 1.36) | .202 |
| Carcinosarcoma & Mixed | 1.00 (0.93, 1.09) | .926 | 0.95 (0.87, 1.04) | .235 |
| Others/NOS | 0.93 (0.84, 1.02) | .102 | 0.88 (0.80, 0.98) | .014 |
| **Grade** |  |  |  |  |
| Well differentiated | Ref |  | Ref |  |
| Moderately differentiated | 1.00 (0.94, 1.05) | .911 | 0.99 (0.93, 1.04) | .627 |
| Poorly differentiated | 1.05 (0.99, 1.11) | .133 | 1.03 (0.96, 1.10) | .467 |
| Unknown | 1.06 (0.98, 1.14) | .147 | 1.05 (0.95, 1.16) | .323 |

Abbreviation: USD, United States dollar

^*^Adjusted to 2023
