# Supplementary material for: Impact of the COVID-19 pandemic on diagnosis, treatment, and prognosis of cervical, endometrial, and ovarian cancers: A retrospective cohort study based on the SEER database
Source: Medicine (Baltimore). 2026 Oct 16;105(42):e51038. doi: 10.1097/MD.0000000000051038 (PMC13681103; doi:10.1097/MD.0000000000051038)
Supplement: Supplementary file 13 [file medi-105-e51038-s014.docx]

Table S9. Predictors of ovarian cancer diagnosis in 2020 (Pandemic cohort) (compared to 2018-2019 [Pre-pandemic cohort]), 17 SEER registries, 2018-2020

| **Characteristic** | **Univariable** | | **Multivariable** | |
| --- | --- | --- | --- | --- |
|  | **OR (95% CI)** | **p-value** | **OR (95% CI)** | **p-value** |
| **Age** |  |  |  |  |
| 20~49 | Ref |  | Ref |  |
| 50~64 | 1.04 (0.94, 1.16) | .460 | 1.05 (0.94, 1.17) | .406 |
| 65~74 | 1.03 (0.92, 1.16) | .573 | 1.04 (0.92, 1.17) | .572 |
| 75~85 | 1.01 (0.88, 1.15) | .904 | 1.00 (0.87, 1.15) | .999 |
| **Race** |  |  |  |  |
| White | Ref |  | Ref |  |
| Black | 1.00 (0.87, 1.16) | .965 | 0.99 (0.86, 1.15) | .926 |
| Other | 1.10 (0.98, 1.24) | .099 | 1.12 (0.99, 1.26) | .073 |
| **Marital status** |  |  |  |  |
| Unmarried | Ref |  | Ref |  |
| Married | 0.96 (0.89, 1.04) | .291 | 0.95 (0.88, 1.03) | .225 |
| **Median household income^*^** |  |  |  |  |
| ＜75,000USD | Ref |  | Ref |  |
| ≥75,000USD | 1.03 (0.94, 1.13) | .522 | 1.06 (0.95, 1.18) | .314 |
| **Rural-Urban continuum** |  |  |  |  |
| Rural | Ref |  | Ref |  |
| Urban | 0.95 (0.83, 1.08) | .427 | 0.91 (0.78, 1.06) | .209 |
| **Stage** |  |  |  |  |
| Localized | Ref |  | Ref |  |
| Regional | 0.96 (0.85, 1.08) | .491 | 0.96 (0.85, 1.09) | .535 |
| Distant | 1.03 (0.93, 1.13) | .611 | 1.01 (0.90, 1.14) | .891 |
| **Histology** |  |  |  |  |
| High-grade serous carcinoma | Ref |  | Ref |  |
| Low-grade serous carcinoma | 1.06 (0.85, 1.33) | .593 | 1.05 (0.80, 1.39) | .722 |
| Endometrioid carcinoma | 0.97 (0.86, 1.11) | .690 | 1.00 (0.83, 1.19) | .959 |
| Clear cell carcinoma | 0.94 (0.81, 1.09) | .404 | 0.91 (0.77, 1.07) | .254 |
| Mucinous carcinoma | 1.02 (0.86, 1.20) | .818 | 1.03 (0.84, 1.26) | .764 |
| Others/NOS | 0.96 (0.87, 1.06) | .380 | 0.94 (0.85, 1.05) | .286 |
| **Grade** |  |  |  |  |
| Well differentiated | Ref |  | Ref |  |
| Moderately differentiated | 0.91 (0.76, 1.08) | .272 | 0.92 (0.76, 1.11) | .369 |
| Poorly differentiated | 0.94 (0.83, 1.06) | .304 | 0.95 (0.79, 1.14) | .584 |
| Unknown | 1.00 (0.88, 1.14) | .971 | 1.03 (0.86, 1.24) | .715 |

Abbreviation: USD, United States dollar

^*^Adjusted to 2023
